# A Naturally Occurring Hypoallergenic Variant of Vespid Antigen 5 from *Polybia scutellaris* Venom as a Candidate for Allergen-Specific Immunotherapy

**DOI:** 10.1371/journal.pone.0041351

**Published:** 2012-07-23

**Authors:** Sabrina E. Vinzón, Cristina Marino-Buslje, Elena Rivera, Mirtha Biscoglio de Jiménez Bonino

**Affiliations:** 1 Departamento de Química Biológica e Instituto de Química y Fisicoquímica Biológicas (IQUIFIB), Facultad de Farmacia y Bioquímica, Universidad de Buenos Aires, Buenos Aires, Argentina; 2 Departamento de Físico-Matemática, Facultad de Farmacia y Bioquímica, Universidad de Buenos Aires, Buenos Aires, Argentina; Centre de Recherche Public de la Santé (CRP-Santé), Luxembourg

## Abstract

Stings by insects from the Hymenoptera order are known to cause life-threatening allergic reactions and impair life quality. Despite the effectiveness of conventional vespid venom immunotherapy, more standardized and safer allergy vaccines are required and recombinant hypoallergenic variants are important clinical tools. Antigen 5 is a major allergen of vespid venoms and it was previously reported that Antigen 5 from *Polybia scutellaris* (Poly s 5) could be a hypoallergenic variant. In this work we assess the immunological behavior and allergenic activity of Poly s 5 in order to explore its suitability for specific immunotherapy. With this aim, recombinant Poly s 5 was expressed in *Pichia pastoris* and the presence of cross-reactive epitopes with Pol a 5, a known allergenic Antigen 5, was investigated both at the IgG and IgE levels, by ELISA assays and a basophil-mediator release assay respectively. A molecular model was also built to better understand the relationship between immunological and structural aspects. In mice, Poly s 5 induced IgG antibodies which cross-reacted with Pol a 5. However, Poly s 5 induced only minimal amounts of IgE and was a poor inducer of basophil-mediator release, even when the cells were sensitized with Pol a 5-specific IgE. Moreover, Poly s 5-specific serum showed a specific protective activity and was able to inhibit the Pol a 5-induced basophil degranulation. Structural analysis from the molecular model revealed that a few amino acid substitutions in the N-terminal region of Poly s 5 should lead to an alteration of the surface topography and electrostatic potential of the epitopes which could be responsible for its hypoallergenic behavior. These findings, taken as a whole, show that Poly s 5 is likely a naturally occurring hypoallergenic Antigen 5 variant.

## Introduction

Allergies are the most common immune-mediated diseases, with a current prevalence of up to 30% in industrialized countries [1]. Specific immunotherapy (SIT), which is based on the administration of increasing doses of allergen extracts to patients, is the only specific and disease-modifying treatment for allergy, causing a long-lasting symptom relief [2]–[4]. SIT involves several immunological mechanisms and it has been pointed out that a successful treatment is associated with certain features. A number of studies indicate that the induction of allergen-specific IgG antibodies plays an important role in allergy vaccination, capturing the allergen before reaching the effector cell-bound IgE and interfering with the IgE-mediated antigen presentation [5].

Stings by insects of the Apidae family (honeybees and bumblebees), those from the Vespidae family (Vespula, Dolichovespula, Vespa and Polistes genera) and, in some regions, also of the Formicidae family (ants), are one of the major causes of severe, generalized, IgE-mediated hypersensitivity reactions that can be fatal [6]. Immunotherapy for vespid allergy is at present carried out with venom extracts (venom immunotherapy or VIT). Although it has been demonstrated that VIT is clinically effective [2], severe and life-threatening anaphylactic side effects may be induced after the administration of crude allergen extracts. Besides, extract–based immunotherapy includes the risk of inducing new sensitizations. These drawbacks have limited the widespread application of VIT. To avoid such effects, the development of modified allergens with reduced allergenicity has been proposed thus leading to their utilization in high doses with a reduced risk of anaphylactic reactions. Moreover, the use of recombinant proteins over natural allergen extracts, allows the administration of a certain amount of the active antigen which can be formulated in a standardized way [7].

Vespid venoms contain three major allergens: phospholipase A_1_, hyaluronidase and Antigen 5 (Ag 5), the latter of still unknown function [8]. New vaccination strategies are being focused on Ag 5 [9], which has been isolated from the venom of all the clinically relevant species. The sting of *Polybia scutellaris*, a South American wasp, does not cause allergic symptoms according to epidemiological studies in the region where this vespid lives [10]. However, it has been proven that its venom contains Ag 5 (Poly s 5) [11], [12]. This protein shows 60.0–77.8% identity with Ag 5 s from other vespids, the highest values corresponding to Polistes Ag 5 s thus reflecting their closely related evolutionary origin. Despite sequence similarity, preliminary studies in mice suggested that Poly s 5 constitutes a hypoallergenic variant. That is why we pursued the recombinant expression of *P. scutellaris* Ag 5 in the yeast *Pichia pastoris*, which was found to have the same immunological behavior than the purified natural protein [13]. As most of the specific antibodies in venom immunized animals are specific for discontinuous epitopes, such immunological behavior should indicate a proper folding of the recombinant protein.

In this work, we assess the immunological behavior and allergenic activity of Poly s 5 in comparison to those of its more allergenic homolog from *Polistes annularis*, Pol a 5. The presence of Pol a 5-cross-reactive epitopes was investigated at the IgG as well as the IgE level.

We discuss the finding that Poly s 5 has lower allergenic activity in Balb/c mice and does not induce cross-linking of Pol a 5-sensitized basophils, though it does generate cross-reactive IgG antibodies (Abs) able to block Pol a 5-induced basophil degranulation. Moreover, we analyze a molecular model of the antigen in the context of these results, in order to extend the current knowledge on the structural features which could contribute to Ag 5 allergenicity.

## Results

### Immunization of Mice with Poly s 5 Induces IgG Abs that React with Pol a 5

**Figure 1:**
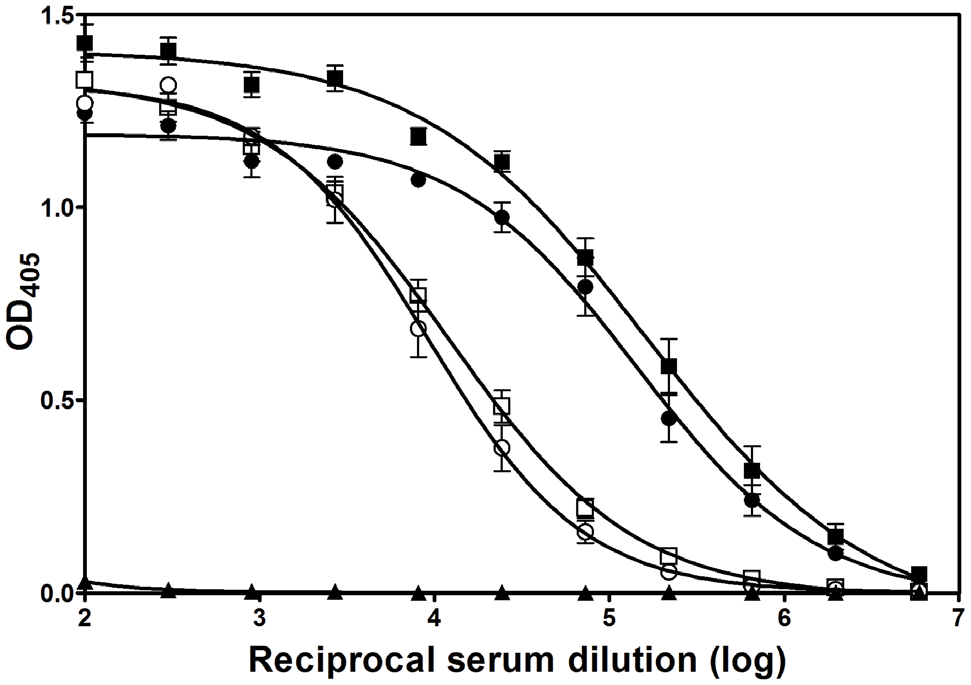
Enzyme immunoassay of Poly s 5- or Pol a 5-specific mouse sera on solid-phase Poly s 5 and Pol a 5. Squares correspond to Poly s 5-specific sera, which were assayed on Poly s 5 (▪) or Pol a 5 (□) as the solid-phase antigen. Pol a 5-specific sera are designated by circles and were assayed on Pol a 5 (•) or Poly s 5 (○) as solid-phase antigen. Closed triangles correspond to the preimmune sera. Mean values ± S.E.M. are shown.

Pol a 5, with a high degree of sequence similarity to Poly s 5, was selected as its allergenic homolog. To search for the presence of cross-reactive B-cell epitopes between Poly s 5 and Pol a 5, specific mouse sera raised against either protein were tested for their binding to solid-phase autologous or homologous protein by direct ELISA (Figure 1). IgG Abs specific for either protein cross-react with each other, as the homologous Ag 5 was recognized by Abs raised against the other Ag 5. Specificity of the IgG cross-reactivity was shown by the lack of reactivity of the control sera. Moreover, both proteins induced comparable specific IgG titers thus showing a similar immunogenicity. The efficiency of autologous and homologous Ag 5 to stimulate a secondary response in mice primed with either Ag 5 was also evaluated (Table 1). Results showed that homologous Ag 5 (groups B and D) was nearly as effective as autologous Ag 5 (groups A and C) in stimulating secondary Ab responses. This provided additional evidence that there is cross-reactivity between Poly s 5 and Pol a 5.

**Table 1:** Poly s 5 and Pol a 5 immunogenicity without adjuvant.

	Immunogen^a^		Antibody titer for primary immunogen^b^		
Group	Primary	Boost	Week 3	Week 5	Week 5/Week 3
A	Poly s 5	Poly s 5	<<1×10^2^	4.3×10^3^	>>50
B	Poly s 5	Pol a 5	<<1×10^2^	4.4×10^2^	>>50
C	Pol a 5	Pol a 5	1.1×10^3^	8.2×10^3^	7.4
D	Pol a 5	Poly s 5	4.7×10^2^	1.6×10^3^	3.5

a Groups of four BALB/c mice were immunized with the primary immunogen at week 0 and week 2 and with the secondary immunogen or boost at week 4.

b Antibody titer represents reciprocal serum dilution for one-third maximal binding to solid phase Ag.

### Allergenicity of Poly s 5

**Figure 2:**
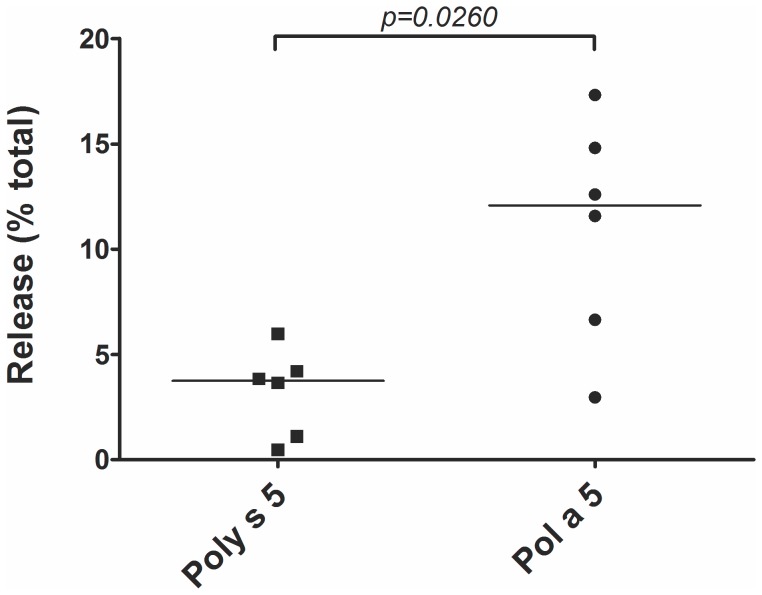
Determination of Poly s 5 allergenic potential. RBL cells were loaded with IgE from mice immunized with Poly s 5 (▪) or Pol a 5 (•) and then exposed to the corresponding autologous Ag 5. Each symbol represents an individual serum. Percentages of β-hexosaminidase release are displayed. Statistical analysis was performed using a non-parametric Mann–Whitney U-test to compare release between the groups.

The allergenic potential of Poly s 5 was analyzed by means of a degranulation assay using the rat basophilic leukemia cell line Rat Basophilic Leukemia (RBL)-2H3 (Figure 2); individual mouse sera were analyzed for their content of Ag 5-specific IgE. As judged by the percentage of β-hexosaminidase release, Poly s 5 induced significantly less specific IgE Abs than Pol a 5, thus showing a reduced allergenicity.

**Figure 3:**
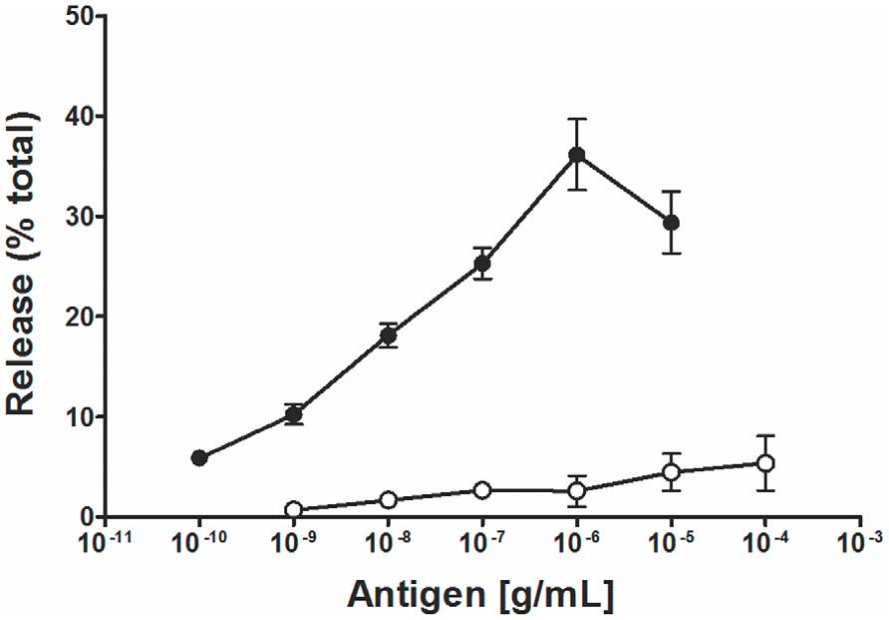
IgE cross-reactivity between Poly s 5 and Pol a 5. RBL cells were loaded with IgE from mice immunized with Pol a 5 and then exposed to increasing concentrations of either Pol a 5 (•) or Poly s 5 (○). Mean percentages of β-hexosaminidase release of duplicates ± S.E.M. are displayed.

In order to evaluate significant cross-reactivity at the IgE level, rat basophils were loaded with IgE from mice immunized with Pol a 5 and then exposed to either Pol a 5 or Poly s 5. Pol a 5 led to a strong dose-dependent β-hexosaminidase release from basophils (Figure 3), with a maximal value at concentrations of 10^−6^ g/mL, whereas Poly s 5 was not able to induce a significant mediator release up to the concentration of 10^−4^ g/mL thus indicating its lesser allergenic activity as well as a low cross-reactivity at the IgE level.

### Protective Capacity of IgG Abs Induced by Poly s 5

**Figure 4:**
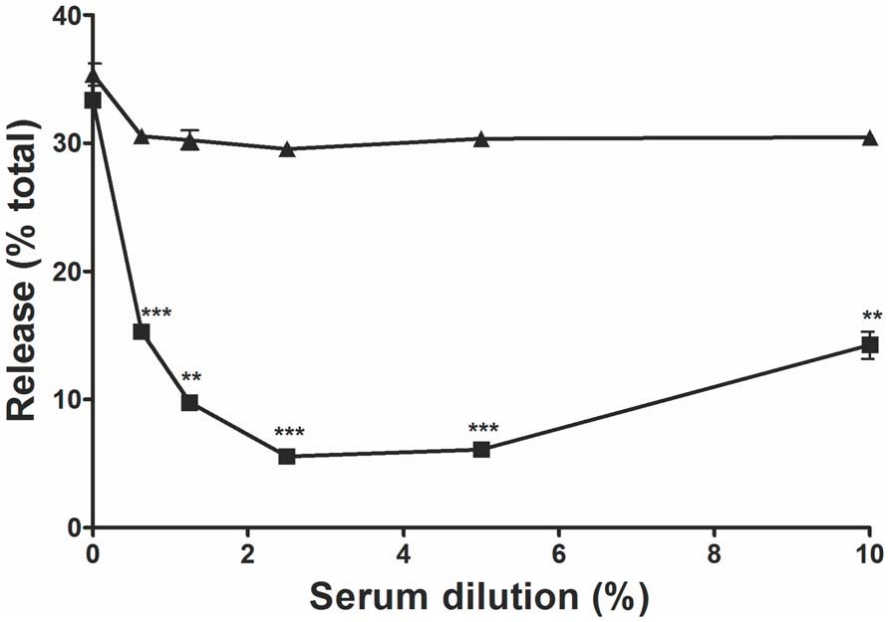
Inhibition of Pol a 5-induced RBL degranulation by Poly s 5-specific Abs. RBL cells were loaded with Pol a 5-specific mouse IgE and then exposed to Pol a 5, which was preincubated with increasing concentrations of mouse Poly s 5-specific serum (▪) or preimmune serum (▴). Mean percentages of β-hexosaminidase release of duplicates ± S.E.M. are displayed. Statistical significance was assessed using an unpaired Student’s t test (**p<0.01; ***p<0.001 vs. preimmune serum).

We also investigated whether IgG Abs induced by immunization with the hypoallergenic Poly s 5 could inhibit Pol a 5-specific basophil degranulation in vitro by RBL cell mediator-release inhibition experiments. Basically, Pol a 5 was preincubated with increasing concentrations of mice anti-Poly s 5 Abs or a control serum and then RBL cells preloaded with Pol a 5-specific IgE were exposed to the immune complexes. Poly s 5-specific Abs significantly inhibited the Pol a 5-induced mediator release from RBL cells (Figure 4), while no inhibition was observed when the allergen was preincubated with the same concentrations of preimmune serum. These results confirm that cross-reactive IgG Abs raised by Poly s 5 are also capable of blocking Pol a 5 binding to effector cell-bound Pol a 5-specific IgE.

### Analysis of the Molecular Model of Poly s 5

A molecular model of Poly s 5 was built by using the known crystallographic structure of Ves v 5 as the template [14]. These proteins share a 59% sequence identity; as expected, their superposition showed a very similar overall structure (RMSD of 0.17 Å for 204 superimposed Cα; Figure S1) and displayed the α-β-α sandwich core fold previously described for the superfamily, maintaining all the secondary structural elements (strands A–D and helices I–IV) [14]. The main structural differences are found in sequence segments 8–10, 19–21, 23–28, 99–101 and 122–127 (Poly s 5 numbering). There is an additional short helix between residues 24 and 27 in Poly s 5 due to a ^25^SSK^27^ insertion when compared to Ves v 5 sequence; this helix was also found when modeling Pol a 5 with the same template.

### Mapping of Putative Cross-reactive Epitopes

**Figure 5:**
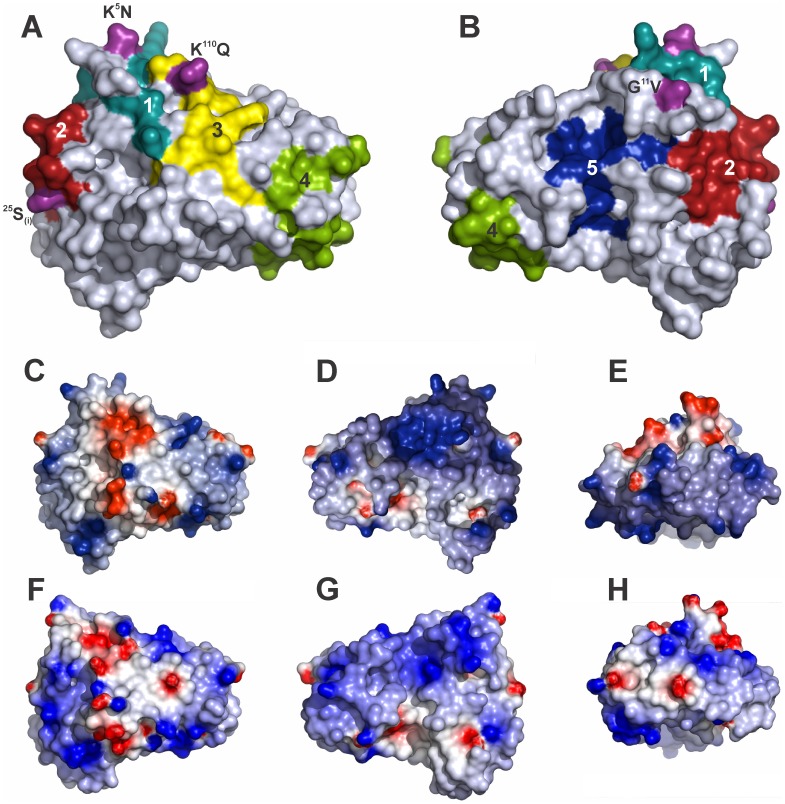
Analysis of the surface of Poly s 5 molecular model. (A and B) Views of Poly s 5 model. In Panel B the molecule is rotated 180° on the y axis with respect to Panel A. Conserved surface patches proposed are numbered and colored. Amino acid changes exclusive for Poly s 5 and located on the molecular surface are in purple and substitutions are indicated by the single-letter amino acid code. The surface electrostatic potential for Poly s 5 (C, D and E) and Pol a 5 (F, G and H) is represented by a blue to red scale corresponding to −5 kT to +5 kT respectively. In panels A, C and F the molecules have the same orientation; the same is true for panels B, D and G. In panels E and H the molecules are oriented to show the red patch.

Ag 5 s from the same genus are serologically cross-reactive and a different degree of cross-reactivity has also been observed between Ag 5 s from different vespid genera, reflecting their high sequence similarity and the phylogenetic relationship between organisms [15], [16]. Structurally conserved allergen molecular surfaces have been identified among different vespid Ag 5 s by Henriksen et al. [14], providing the structural basis for the observed allergic cross-reactivity. Five conserved surface patches, proposed as putative cross-reactive epitopes, were revealed on the Ves v 5 structure. These epitope candidates were mapped onto the surface of the Poly s 5 model (Figure 5, Panels A and B).

**Figure 6:**
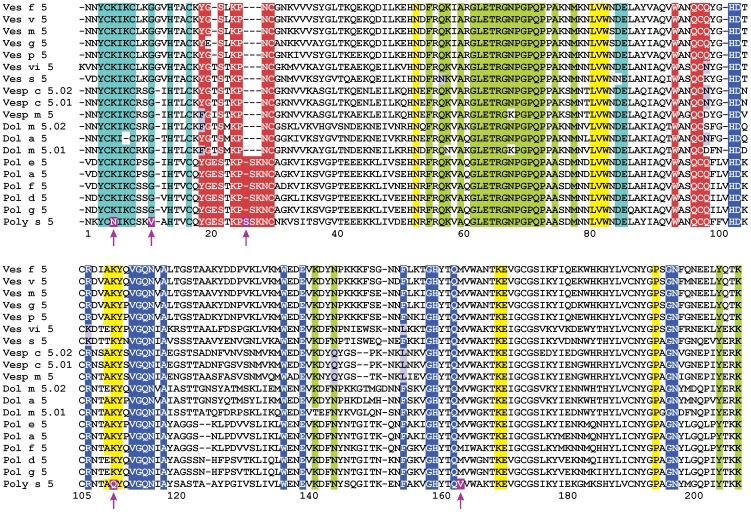
Sequence alignment of vespid Ag 5 s. From top to bottom: Ves f 5, *Vespula flavopilosa* Ag 5 (P35783); Ves v 5, *V. vulgaris* Ag 5 (Q05110); Ves m 5, *V. maculifrons* Ag 5 (P35760); Ves g 5, *V. germanica* Ag 5 (P35784); Ves p 5, *V. pensylvanica* Ag 5 (P35785); Ves vi 5, *V. vidua* Ag 5 (P35787); Ves s 5, *V. squamosa* Ag 5 (P35786); Vesp c 5.02, *Vespa crabro* Ag 5 (P35782); Vesp c 5.01, *V. crabro* Ag 5 (P35781); Vesp m 5, *V. mandarinia* Ag 5 (P81657); Dol m 5.01, *Dolichovespula maculata* Ag 5 (P10736); Dol a 5, *D. arenaria* Ag 5 (Q05108); Dol m 5.02, *D. maculata* Ag 5 (P10737); Pol e 5, *Polistes exclamans* Ag 5 (P35759); Pol a 5, *P. annularis* Ag 5 (Q05109); Pol f 5, *P. fuscatus* Ag 5 (P35780); Pol d 5, *P. dominulus* Ag 5 (P81656); Pol g 5, *P. gallicus* Ag 5 (P83377); Poly s 5, *Polybia scutellaris* Ag 5 (Q7Z156). Fully conserved residues of the five (#1–5) proposed epitopes are on cyan, red, yellow, green and blue background respectively. Residues which are non-conserved or conservatively substituted are on white and gray background respectively. Residues exclusively changed in Poly s 5 are on purple background and marked with arrows. Numbering corresponds to the Poly s 5 sequence.

Sequence alignment of vespid Ag 5 s with a proven cross-reactivity (Figure 6) showed that of the 85 conserved residues, of which four are changed only in Poly s 5: Asn^5^Lys, Val^11^Gly, Gln^110^Lys and Val^163^Met (Poly s 5 numbering). Additionally, a Ser insertion is present at position 25. Interestingly, 4 of these 5 changes are located in the conserved patches mentioned above (Figure 5). The cyan patch (patch #1) has a conserved surface of about 540 Å^2^ and it harbors two of the changes: Asn^5^Lys and Val^11^Gly, while the yellow patch (#3; 460 Å^2^) has the Gln^110^Lys substitution. The red one (#2; 660 Å^2^) contains the ^25^Ser insertion. Instead, the green and blue patches (#4 and #5; 1050 and 410 Å^2^ respectively) are perfectly conserved on Poly s 5 surface. As appreciated in Figure 6, putative epitopes are formed by non-contiguous residues. As regards red and cyan epitope candidates, analysis of their structure shows that they distinctly cover the N-terminal segment. When the surface electrostatic potential of Poly s 5 is compared with that of Pol a 5 in the area containing the putative epitopes (Figure 5, Panels C-H), some drastic changes are revealed in the regions of Asn^5^Lys and Gln^110^Lys substitutions; a blurring of the electropositive charge is observed, as expected from the substitution of a positively charged residue (Lys) for a neutral one (Asn or Gln). Moreover, a large disturbance of surface potential was observed in the region surrounding ^25^Ser insertion; a more detailed inspection also reveals a great change of the epitope topography. Furthermore, when values of solvent accessible surface area per residue were compared and analyzed in the alignment of Poly s 5 and Pol a 5 sequences, it became evident that ^25^Ser insertion in Poly s 5 modifies solvent exposure of several neighboring residues (Figure S2), thus contributing to the loss of complementarity in this region.

## Discussion

Vespid venom allergen Ag 5 has been characterized in great detail from various species and several Ag 5 s have been cloned. In this study we report an in-depth characterization of the first naturally occurring Ag 5 hypoallergenic variant, Poly s 5. We also explore the features that can make it suitable as a vaccine for safer immunotherapy, replacing the widely used non-standardized natural venom extracts. Two important requirements must be fulfilled: (i) strong immunogenicity; (ii) the presence of B-cell epitopes shared with the sensitizing allergen to induce blocking Abs and hence be successful [17]. When investigating the immune response elicited by Poly s 5 in mice we found that it induces a robust Poly s 5-specific IgG response, including IgG Abs that cross-react with Pol a 5, a clinically relevant allergen. Moreover, those Abs showed a specific protective activity and inhibited the Pol a 5-induced basophil degranulation when tested in RBL assays. It is important to point out that hypoallergens which induce a robust IgG response in BALB/c mice will also likely induce blocking IgG Abs in humans [18].

The risk of using allergenic extracts in immunotherapy makes the development of new strategies such as the use of hypoallergenic variants relevant. We found that Poly s 5, even when administered with a strong adjuvant, induces a lower IgE level upon immunization of mice thus exhibiting reduced allergenicity *in vivo*. On the other hand, Poly s 5 induces a weaker mediator release than Pol a 5 in assays with Pol a 5-sensitized RBL cells (Figure 3), thus indicating that it also displays low cross-reactivity with this allergen at the IgE level.

We undertook a comparative study of structural aspects of Poly s 5 against those of Ves v 5 to contribute to the understanding of the structural requirements for Ag 5 allergenicity. Ag 5 s sequence alignment has shown a high degree of similarity supporting a common protein fold [19]. Additionally, such similarity allows the existence of conserved surface areas that constitute candidates for epitopes defined by cross-reactive antibodies [20]. Cross-reactivity between members of the Ag 5 family is well established, and five highly conserved regions on the surface of Ves v 5 were proposed as putative epitopes.

Antigen-antibody interaction studies indicate that the surface area buried in an epitope is about 600–900 Å^2^ [21]; reduction of this area might cause a decrease in the Ab affinity constant. Major factors determining binding affinity are a perfect fit in the topography of the complementary surfaces of the epitope/paratope interaction [21], as well as the electrostatic complementarity of the interacting surfaces [22]. Our analysis of the molecular model of Poly s 5 shows that only the green and blue patches, as defined by Henriksen et al. [14], are conserved in Poly s 5. However, based on sequence conservation and structural characteristics, the blue patch has been proposed to be a cavity implied in the yet unknown Ag 5 activity, thus not the best candidate for a cross-reactive epitope [14]. Therefore, it is likely that the green patch would be at least partially responsible for the cross-reactivity between Poly s 5 and Pol a 5. On the other hand, the non-conserved residues in the cyan, red and yellow putative epitopes clearly contribute to differences in the local molecular surface and the electrostatic potential which could affect the interaction with antibodies. Analysis of known IgE epitopes indicates that the propensity of Lys being in an epitope is significantly higher than in any other protein-protein interface [23]; moreover, the substitution of even a single Lys can cause an electrostatic disturbance and abolish IgE binding [24], [25]. It is possible that the presence of Lys 5 and 106 (Ves v 5 numbering) is important for IgE binding and their absence plays a role in the lack of allergenicity of Poly s 5.

King et al. [26] have reported that the dominant B cell epitopes of Ves v 5 are in the N-terminal region. Three of the 4 surface-exposed residue changes in Poly s 5 are located in the protein N-terminal area (Asn 5, Val 11, and Ser 25), substantially changing the red and cyan patches properties. The cyan patch has two substitutions, including the mentioned Asn^5^Lys, whereas the insertion of ^25^Ser in the red patch drastically modifies its shape and electrostatic potential. It is interesting to highlight that allergic cross-reactivity requires the cross-linking of receptor bound IgE Abs on the surface of a mast cell or basophil through simultaneous interaction with two conserved surface areas of the protein [20]. This implies that, in order to detect cross-reactivity by the RBL-cell assay, at least two shared cross-reactive epitopes are needed. Our molecular model of Poly s 5 supports the lack of IgE cross-reactivity given that only one putative epitope is conserved. Future studies will include the construction of mutants involving the aforementioned positions to test this hypothesis.

Reduction of IgE binding by substituting highly solvent exposed key amino acids located on conserved surface areas, together with the use of a universal cross-reactive reagent, have been proposed for immunotherapy related to vespid allergens [14], [27]. The Ag 5 from *P. scutellaris*, whose structural and immunological features are described herein, is a naturally occurring variant with minimal allergenic activity but a strong immunogenicity and IgG cross-reactivity with allergenic homologs. Taken as a whole, epidemiological studies and results obtained herein lead us to suggest that Poly s 5 could be a promising candidate for a new strategy of specific immunotherapy for vespid venom allergy.

## Materials and Methods

### Ethics Statement

All animal experimental procedures were performed in accordance to the Argentinean National Research Council Directive RD_N° 1047/05 for ethical biomedical research in laboratory animals. The ethics committee on the use and care for laboratory animals at the Animal House of the School of Exact and Natural Sciences (University of Buenos Aires) approved all the procedures used in this study.

### Allergens

Recombinant Poly s 5 and Pol a 5 were produced in *P. pastoris* and purified as previously described [13]. A plasmid to express the recombinant Pol a 5 in *P. pastoris* was kindly provided by Dr. T. P. King [26].

### Immunization Protocols

Groups of 6 male BALB/c mice were immunized i.p. 5 times at 2-week intervals with 0.2 mL of 10 µg/mL immunogen (Poly s 5 or Pol a 5) with 5 mg/mL alum (ImjectAlum®, Pierce Chemical Company, Rockford, IL, USA) in PBS. One group was immunized only with PBS as a control. Mice were bled 1 week after the last injection and sera were collected.

For measurement of *in vivo* immunogenicity, groups of 4 male BALB/c mice were immunized i.p. twice at a 2-week interval with 0.2 mL of 10 µg/mL of the primary immunogen (Poly s 5 or Pol a 5) without adjuvant. After 2 weeks, mice were injected with 0.2 mL of 10 µg/mL of either the primary immunogen or the homologous Ag 5 as a boost. Sera were collected 1 week after the second and third injections.

### Measurement of Specific and Cross-reactive IgG Antibodies by ELISA

Microtiter plates (Immuno96 MicroWell™ Plates Nunc MaxiSorp™) were coated overnight at 4°C with Poly s 5 or Pol a 5 (2 µg/mL in 0.05 M Tris-HCl buffer, pH 8.0) and thereafter blocked for 1 h with TBS containing 0.05% Tween-20 and 0.5% BSA. Duplicate wells were then incubated overnight at 4°C with mouse serum diluted in the incubation buffer (TBS containing 0.05% Tween 20 and 0.25% BSA). After washing, bound specific mouse IgG was detected with biotinylated goat anti-mouse IgG and an avidin-alkaline phosphatase conjugate (Invitrogen, San Diego, CA, USA). Color development was performed by addition of p-nitrophenyl phosphate (Sigma, St Louis, MO, USA) for 30 min, and absorbance at 405 nm was measured. Curves were fitted by nonlinear regression with GraphPad Prism 5.0 (GraphPad Software, San Diego, CA, USA).

### Rat Basophilic Leukemia (RBL) Cell Mediator-release Experiments

The allergenic potential of each Ag 5 was studied by the RBL assay [28]. Briefly, RBL-2H3 cells (ATCC CRL-2256™, Rockville, MD, USA) were plated in 96-well flat-bottomed tissue culture plates (4×10^4^/well; BD Falcon™, BD Biosciences, San Jose, CA, USA) and passively sensitized for 2 h with mice sera diluted 1∶50. After washing the cells in Tyrode’s buffer (137 mM NaCl, 2.7 mM KCl, 0.5 mM MgCl_2_, 1.8 mM CaCl_2_, 0.4 mM NaH_2_PO_4_, 5.6 mM D-glucose, 12 mM NaHCO_3_, 10 mM HEPES and 0.1% BSA, pH 7.2), cross-linking of the FcεR-bound IgE was induced by adding 100 ng/mL Ag 5 in Tyrode’s buffer for 1 h at 37°C. Spontaneous release was measured by incubation of sensitized RBL-2H3 cells with Tyrode’s buffer and total release was obtained after addition of 1% Triton X-100 (Sigma Chemical Co., St. Louis, MO, USA) to the medium. β -hexosaminidase release into the supernatant was measured by enzymatic cleavage of p-nitro-N-acetyl-β-D-glucosaminide (1.3 mg/mL in 50 mM citrate buffer, pH 4.5; Sigma). After 1-h substrate incubation, the reaction was stopped by addition of 0.4 M glycine buffer, pH 10.5, and the absorbance at 405 nm was determined. Results are expressed as percentage of the total release minus that of the spontaneous release.

The same protocol was utilized for determination of cross-reactivity at the IgE level, although serial dilutions (10^−10^–10^−4^ g/mL) of the cross-linking autologous or homologous Ag 5 were used.

The ability of Poly s 5-specific serum to inhibit Pol a 5-induced degranulation of RBL cells was determined as follows. Pol a *5* (0.01 µg/mL) was preincubated with different dilutions (0, 2.5, 5, and 10%) of mouse anti-Poly s 5, or, as a control, of a preimmune mouse serum, in Tyrode’s buffer for 2 h at 37°C. The mixtures were then exposed to RBL cells passively sensitized with Pol a 5-specific mouse IgE. Mediator-release analysis was performed as described above.

### Sequence Alignment and Molecular Modeling

Ag 5 sequences were retrieved from UniProtKB. Multiple sequence alignment was performed with CLUSTAL X [29] and adjusted manually. Models were built by using the crystal structure of *Vespula vulgaris* Ag 5 (Ves v 5, PDB 1QNX) as the template and the program suite Modeller 9v2 [30]. The stereochemical quality of the models was checked using PROCHECK [31] and Verify3D [32]. All graphical molecular representations were generated using PyMol (DeLano Scientific LLC, San Carlos, CA, USA, http://www.pymol.org). The template and models alpha carbon backbones were superimposed and the RMSD was calculated with the program SWISS-PDB Viewer [33]. Electrostatic potentials were calculated with Delphi [34]. The inner and outer dielectric constants applied to the protein and the solvent, were fixed at 4.0 and 80.0 respectively, and calculations were performed keeping a 0.15 M ionic strength. Values of solvent accessible surface area were obtained with the program MOLMOL [35]; a radius of 1.4 Å for the water molecule was assumed.

## Supporting Information

Figure S1 **Superimposition of Ves v 5 (orange) and modeled Poly s 5 (purple) structures.** Molecules are shown in the ribbon representation. Dashed lines comprise the five regions (a–e) with the worst adjustment: residues 8–10 (a), 19–21 (b), 23–28 (c), 99–101(d) and 122–127(e) corresponding to Poly s 5 numbering.
(TIFF)

Figure S2 **Solvent accessible surface area per residue.** It was calculated for Poly s 5 (dark gray line) and Pol a 5 (orange line) models. Values represent the percentage of solvent exposed area for each individual residue, compared to that of a Gly-X-Gly tripeptide. Dashed line represents the limit above which a residue is considered as exposed (30% exposure). Values are aligned with the corresponding alignment of both proteins. Colored areas represent the conserved surface patches shown in Figures 5 and 6.
(TIFF)

## References

[1] Traidl-Hoffmann C, Jakob T, Behrendt H (2009). Determinants of allergenicity.. J Allergy Clin Immunol.

[2] Golden DB (2005). Insect sting allergy and venom immunotherapy: a model and a mystery.. J Allergy Clin Immunol 115: 439–447; quiz 448.

[3] Larché M, Akdis CA, Valenta R (2006). Immunological mechanisms of allergen-specific immunotherapy.. Nat Rev Immunol.

[4] Niederberger V (2009). Allergen-specific immunotherapy.. Immunol Lett.

[5] Aalberse R (2011). The role of IgG antibodies in allergy and immunotherapy.. Allergy.

[6] Müller U, Golden DB, Lockey RF, Shin B (2008). Immunotherapy for hymenoptera venom hypersensitivity.. Clin Allergy Immunol.

[7] Valenta R, Niederberger V (2007). Recombinant allergens for immunotherapy.. J Allergy Clin Immunol.

[8] King TP, Spangfort MD (2000). Structure and biology of stinging insect venom allergens.. Int Arch Allergy Immunol.

[9] Winkler B, Bolwig C, Seppala U, Spangfort MD, Ebner C (2003). Allergen-specific immunosuppression by mucosal treatment with recombinant Ves v 5, a major allergen of Vespula vulgaris venom, in a murine model of wasp venom allergy.. Immunology.

[10] Amaral V. Venenos de Himenópteros de Argentina: estructura y antigenicidad; 1996; XVI National Meeting on Allergy and Immunology; Buenos Aires, Argentina..

[11] Cascone O, Amaral V, Ferrara P, Vita N, Guillemot JC (1995). Purification and characterization of two forms of antigen 5 from polybia scutellaris venom.. Toxicon.

[12] Pirpignani ML, Rivera E, Hellman U, Biscoglio de Jiménez Bonino M (2002). Structural and immunological aspects of Polybia scutellaris Antigen 5.. Arch Biochem Biophys.

[13] Vinzón SE, Pirpignani ML, Nowicki C, Biscoglio de Jiménez Bonino M (2010). Molecular cloning and expression in Pichia pastoris of a hypoallergenic antigen 5.. Protein Expr Purif.

[14] Henriksen A, King TP, Mirza O, Monsalve RI, Meno K (2001). Major venom allergen of yellow jackets, Ves v 5: structural characterization of a pathogenesis-related protein superfamily.. Proteins.

[15] Bilo BM, Rueff F, Mosbech H, Bonifazi F, Oude-Elberink JN (2005). Diagnosis of Hymenoptera venom allergy.. Allergy.

[16] Hoffman DR (1993). Allergens in Hymenoptera venom. XXV: The amino acid sequences of antigen 5 molecules and the structural basis of antigenic cross-reactivity.. J Allergy Clin Immunol.

[17] Wachholz PA, Durham SR (2004). Mechanisms of immunotherapy: IgG revisited.. Curr Opin Allergy Clin Immunol.

[18] Niederberger V, Horak F, Vrtala S, Spitzauer S, Krauth MT (2004). Vaccination with genetically engineered allergens prevents progression of allergic disease.. Proc Natl Acad Sci U S A.

[19] Chothia C, Finkelstein AV (1990). The classification and origins of protein folding patterns.. Annu Rev Biochem.

[20] Aalberse RC, Akkerdaas J, van Ree R (2001). Cross-reactivity of IgE antibodies to allergens.. Allergy.

[21] Davies DR, Padlan EA, Sheriff S (1990). Antibody-antigen complexes.. Annu Rev Biochem.

[22] Van Oss CJ (1995). Hydrophobic, hydrophilic and other interactions in epitope-paratope binding.. Mol Immunol.

[23] Oezguen N, Zhou B, Negi SS, Ivanciuc O, Schein CH (2008). Comprehensive 3D-modeling of allergenic proteins and amino acid composition of potential conformational IgE epitopes.. Mol Immunol.

[24] Gafvelin G, Parmley S, Neimert-Andersson T, Blank U, Eriksson TL (2007). Hypoallergens for allergen-specific immunotherapy by directed molecular evolution of mite group 2 allergens.. J Biol Chem.

[25] Gehlhar K, Rajashankar KR, Hofmann E, Betzel C, Weber W (2006). Lysine as a critical amino acid for IgE binding in Phl p 5b C terminus.. Int Arch Allergy Immunol.

[26] King TP, Jim SY, Monsalve RI, Kagey-Sobotka A, Lichtenstein LM (2001). Recombinant allergens with reduced allergenicity but retaining immunogenicity of the natural allergens: hybrids of yellow jacket and paper wasp venom allergen antigen 5 s.. J Immunol.

[27] King TP, Lu G, Gonzalez M, Qian N, Soldatova L (1996). Yellow jacket venom allergens, hyaluronidase and phospholipase: sequence similarity and antigenic cross-reactivity with their hornet and wasp homologs and possible implications for clinical allergy.. J Allergy Clin Immunol.

[28] Hoffmann A, Vieths S, Haustein D (1997). Biologic allergen assay for in vivo test allergens with an in vitro model of the murine type I reaction.. J Allergy Clin Immunol.

[29] Thompson JD, Gibson TJ, Plewniak F, Jeanmougin F, Higgins DG (1997). The CLUSTAL_X windows interface: flexible strategies for multiple sequence alignment aided by quality analysis tools.. Nucleic Acids Res.

[30] Sali A, Blundell TL (1993). Comparative protein modelling by satisfaction of spatial restraints.. J Mol Biol.

[31] Laskowski RA, MacArthur MW, Moss DS, Thornton JM (1993). PROCHECK: a program to check the stereochemical quality of protein structures.. J Appl Cryst.

[32] Eisenberg D, Luthy R, Bowie JU (1997). VERIFY3D: assessment of protein models with three-dimensional profiles.. Methods Enzymol.

[33] Guex N, Peitsch MC (1997). SWISS-MODEL and the Swiss-PdbViewer: an environment for comparative protein modeling.. Electrophoresis.

[34] Nicholls A, Honig B (1991). A rapid finite difference algorithm, utilizing successive over-relaxation to solve the Poisson–Boltzmann equation.. J Comp Chem.

[35] Koradi R, Billeter M, Wuthrich K (1996). MOLMOL: a program for display and analysis of macromolecular structures.. J Mol Graph 14: 51–55, 29–32.

