# Supplementary figures and images for: A Naturally Occurring Hypoallergenic Variant of Vespid Antigen 5 from Polybia scutellaris Venom as a Candidate for Allergen-Specific Immunotherapy
Source: PLoS One. 2012 Jul 23;7(7):e41351. doi: 10.1371/journal.pone.0041351 (PMC3402526; doi:10.1371/journal.pone.0041351)

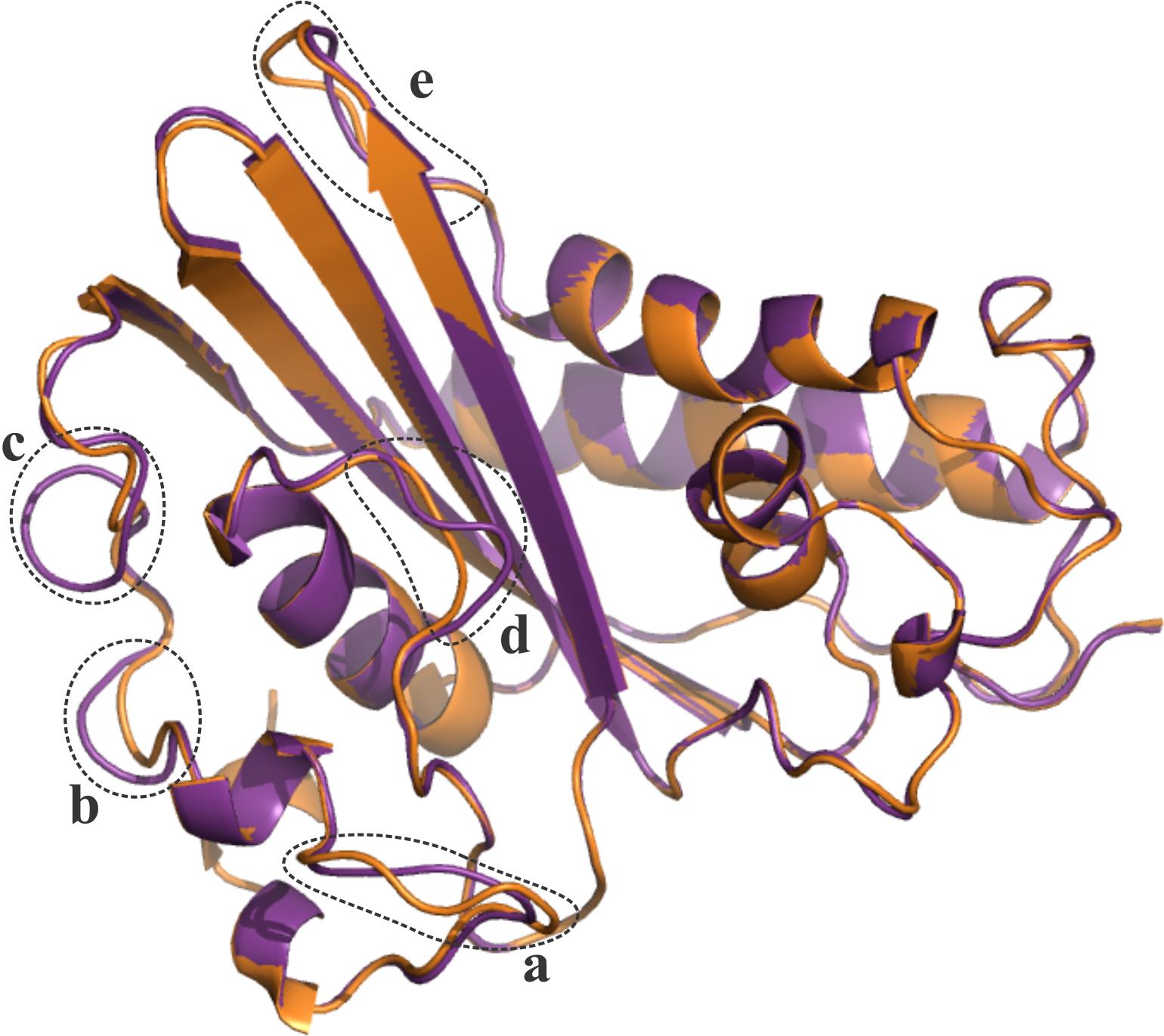

Supplement: Figure S1 — Superimposition of Ves v 5 (orange) and modeled Poly s 5 (purple) structures. Molecules are shown in the ribbon representation. Dashed lines comprise the five regions (a–e) with the worst adjustment: residues 8–10 (a), 19–21 (b), 23–28 (c), 99–101(d) and 122–127(e) corresponding to Poly s 5 numbering. (TIFF) [file pone.0041351.s001.tiff]

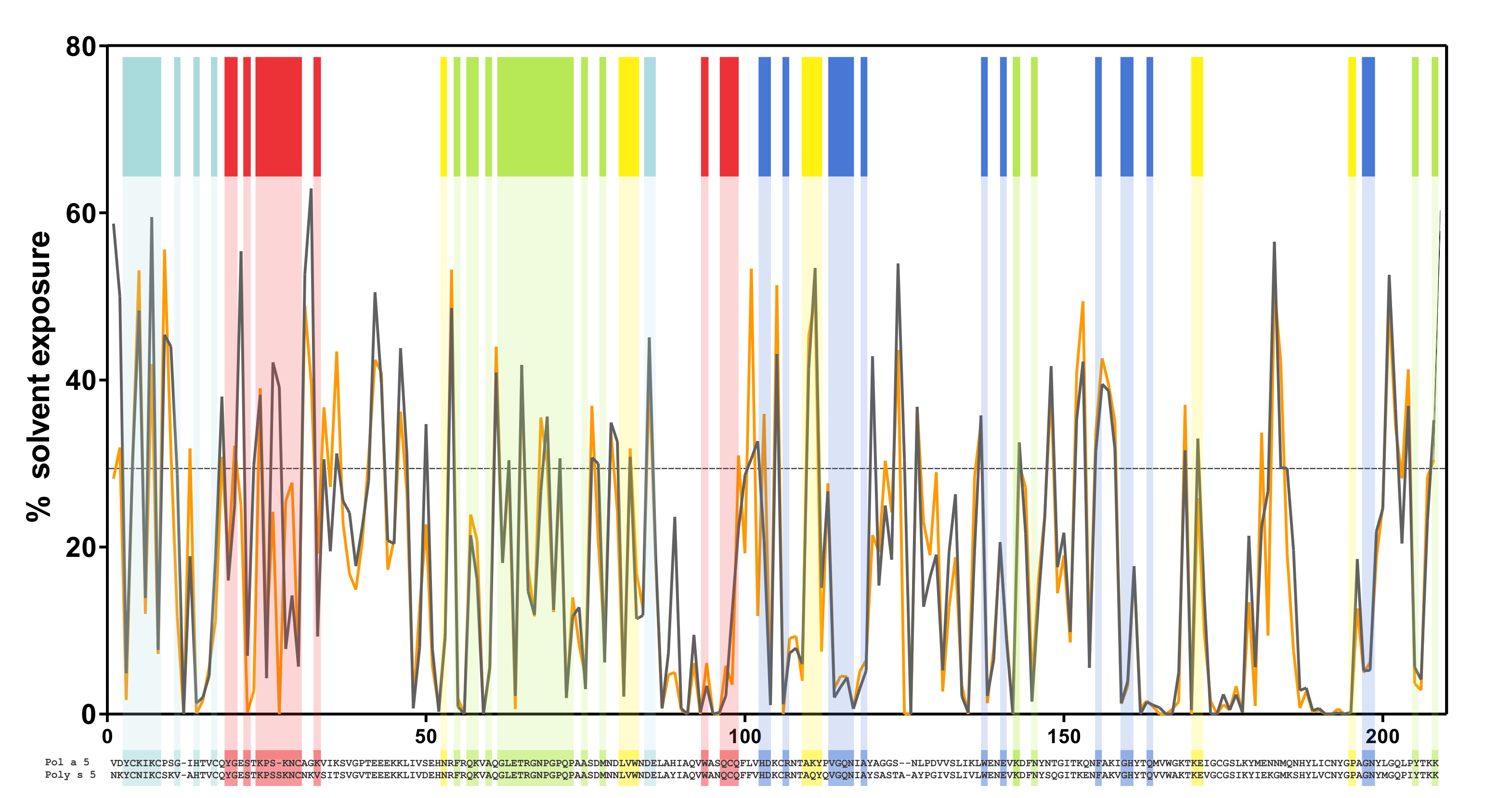

Supplement: Figure S2 — Solvent accessible surface area per residue. It was calculated for Poly s 5 (dark gray line) and Pol a 5 (orange line) models. Values represent the percentage of solvent exposed area for each individual residue, compared to that of a Gly-X-Gly tripeptide. Dashed line represents the limit above which a residue is considered as exposed (30% exposure). Values are aligned with the corresponding alignment of both proteins. Colored areas represent the conserved surface patches shown in Figures 5 and 6. (TIFF) [file pone.0041351.s002.tiff]
